# Retraction: The yield of procalcitonin and Interleukin-6 in predicting intraamniotic infection in the presence of intrapartum fever: A pilot study

**DOI:** 10.1371/journal.pone.0339075

**Published:** 2025-12-18

**Authors:** The PLOS One Editors

The *PLOS One* Editors retract this article [1] because it was identified as one of a series of submissions for which we have concerns about compromised peer review.

The Editors have no evidence of any author involvement in the peer review concerns.

ZE, HA, IG, and OR did not agree with the retraction. SM and SGG either did not respond directly or could not be reached.
